# A rare giant gastric trichobezoar in a young female patient: Case report and review of the literature

**DOI:** 10.1002/ccr3.5152

**Published:** 2021-12-11

**Authors:** Dimitra G. Delimpaltadaki, Ioannis G. Gkionis, Mathaios E. Flamourakis, Andreas F. Strehle, Emmanouil N. Bachlitzanakis, Michail I. Giakoumakis, Manousos S. Christodoulakis, Konstantinos G. Spiridakis

**Affiliations:** ^1^ Department of General Surgery Venizeleio General Hospital Crete Greece

**Keywords:** bezoar, gastroenterology, general surgery, Rapunzel syndrome, trichobezoar

## Abstract

A bezoar is an aggregate of undigested foreign materials that accumulate in the gastrointestinal tract and may cause serious symptoms or even life‐threatening complications. Trichobezoars, a subtype of bezoars, are a rare condition usually occurring in females with psychiatric disorders, with Rapunzel syndrome being an uncommon form of trichobezoar.

## Graphical abstract

Trichobezoars of the gastrointestinal tract are a rare entity but may pose a potentially serious health problem. Physicians must be aware of the risk factors of trichobezoars’ formation and the clinical findings that may facilitate diagnosis.

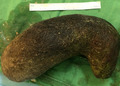

## 1 INTRODUCTION

A bezoar constitutes a solid mass of poorly digested or undigested materials which accumulate in the gastrointestinal (GI) tract and represents an uncommon pathological entity. Throughout history bezoars developing in animals were used as antidotes against snakebite, epilepsy, and even the plague.^1^ To this day, they constitute a part of the traditional Chinese medicine.^2^

Bezoars are categorized into 4 groups according to their composition.^3^ (A) Phytobezoars are composed of indigestible material in fruits and vegetables, (B) Trichobezoars are mainly an accumulation of a patient's hair, although hair from animals, carpets, or toys are occasionally recovered, (C) Pharmacobezoars consist of various undigested medicines, and, finally, (D) Other bezoars constitute a heterogeneous group which includes aggregates from a variety of substances such as pieces of paper, gloves, shellac, styrofoam, cement, milk curd, and several other materials.^3^ An uncommon form of trichobezoar is known as the Rapunzel syndrome.^2^

Bezoars are usually asymptomatic.^1^, ^2^, ^3^ As the size of a bezoar increase, symptoms begin to develop.^1^, ^2^, ^3^ In the rare case of a complete intestinal obstruction or perforation, a patient may present with symptoms of acute abdomen.^1^, ^2^, ^3^

We report a case of a trichobezoar occupying the entire stomach in a 15‐year‐old female patient. This report aims to further educate clinical doctors on this rare entity which can have fatal consequences for the patient.

## 2 CASE PRESENTATION

We present the case of a 15‐year‐old Caucasian female patient who was admitted to the hospital with symptoms of vomiting, epigastric pain, weight loss, progressive postprandial abdominal discomfort, and nausea. All symptoms were relieved after vomiting. On inspection, she was ill looking and pale, as well as dehydrated, while, based on her body weight, she was falling on the lower limit of the growth curve (Weight = 46kgr, Height = 150 cm). Her vital signs on admission were the following: Temperature = 36.9°C, Heart Rate = 83, Respiratory Rate = 21, and Blood Pressure = 98/59 mmHg. The patient was taking no medication and had no other underlying medical condition or intellectual disability. Furthermore, she had no history of previous surgical interventions.

The clinical examination revealed halitosis, a mobile palpable mass occupying the region between the umbilicus and the xiphoid process with size 25 × 9 cm, normal bowel sounds, and no muscle contraction of the abdominal wall or any other signs indicative of peritonitis. In addition, there was no blood or palpable mass on digital rectal examination. No abnormal signs were found during physical examination of the cardiopulmonary and urogenital systems.

The blood tests revealed anemia (Hemoglobin concentration = 7.1 g/dl with normal values ranging between 11.9 and 14.7 g/dl and Hematocrit = 25.1% with normal values ranging between 36.8% and 45.6%). All other blood test results were falling within normal range (Table 1).

**TABLE 1:** Blood test results on admission

Blood test markers	On admission	Normal value range
Hb	7.1	11.9–14.7 g/dl
Hct	25.1	36.8–45.6%
WBC	9.200	3.800–10.500/ml
PLT	249.000	150,000–400.000 K/μl
UR	19	8–50 mg/dl
Cr	0.63	0.6–1.4 mg/dl
SGOT	15	10–40 U/L
SGPT	11	10–35 U/L
GGT	7	0–30 U/L
Na^+^	141	136–145 mmol/L
K^+^	4.6	3.5–5.1 mmol/L
CRP	0.4	<0.5 mg/dl

Abbreviations: Cr, Creatinine; CRP, C‐reactive protein; GGT, Gamma glutamyl transferase; Hb, Hemoglobin; Hct, Hematocrit; K^+^, Potassium; Na^+^, Sodium; PLT, Platelets; SGOT, Serum glutamic oxaloacetic transaminase; SGPT, Serum glutamic pyruvic transaminase; UR, Urea; WBC, White blood cells.

**FIGURE 1:**
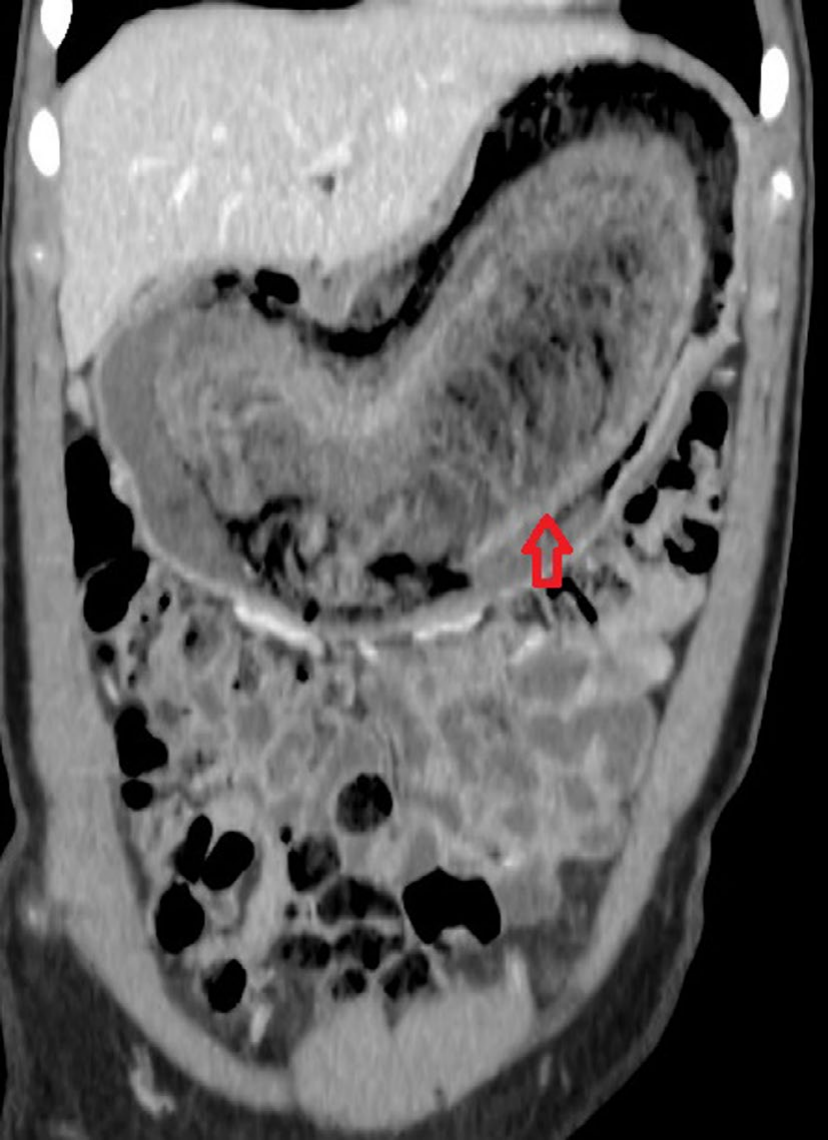
CT scan showing a low‐density intragastric mass which contains air bubbles and exhibits a characteristic mottled appearance. The arrow is demonstrating the intragastric mass

On admission, an abdominal X‐ray was performed, but it was not diagnostic, followed by an abdominal ultrasound (U/S) which revealed a well‐defined mass with dimensions 26cm × 9cm × 9cm in the epigastric region. To determine its origin and composition, an abdominal computerized tomography (CT) was ordered. The imaging results were in accordance with those of U/S, demonstrating the presence of a large, well‐circumscribed, non‐homogeneous mass, which lacked blood supply and occupied the whole stomach. The dimensions of that mass were 26,6cm × 9,7cm × 9,8cm. The images were strongly suggestive of a bezoar (Figure 1).

**FIGURE 2:**
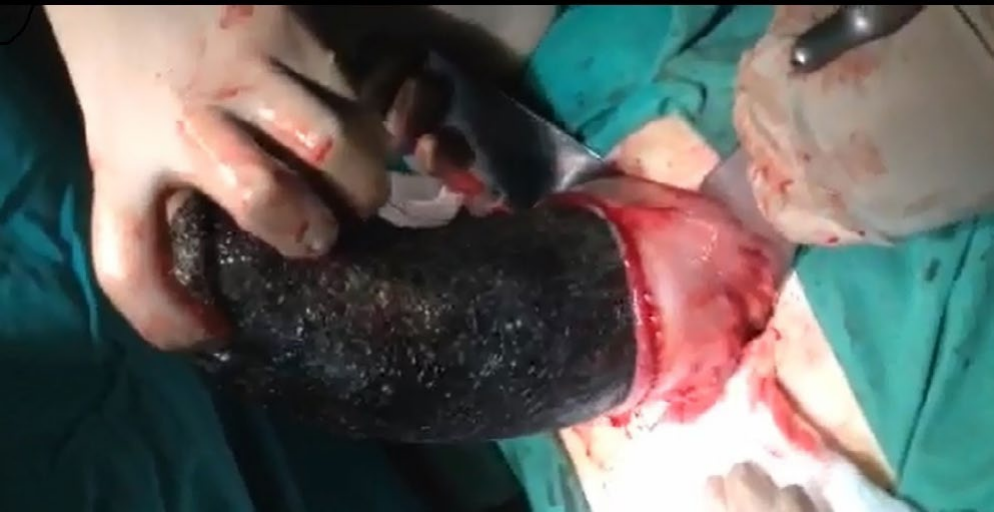
Intraoperative photograph of trichobezoar's subtraction through the gastrotomy

**FIGURE 3:**
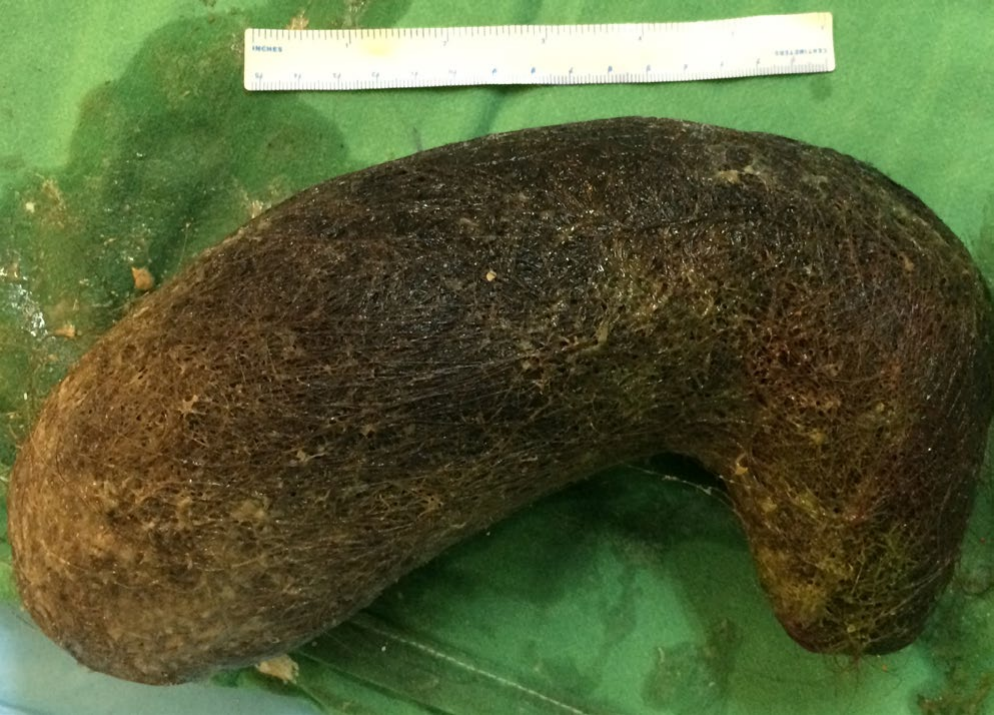
Trichobezoar after its removal from the stomach, with a 15 cm length ruler

An upper GI endoscopy was performed on the second day after admission, confirming the diagnosis of trichobezoar, but failing to extract the mass of hairs. The patient underwent an exploratory laparotomy with an upper midline incision on the same day. Intraoperatively, the diagnosis of trichobezoar was confirmed. An intraluminal mass was seen and felt inside the stomach. A 10 cm incision was made in the anterior wall of the gastric antrum, and a massive trichobezoar in the shape of the stomach with size 27cm × 10cm × 10cm was extracted (Figures 2 and 3).

The patient had an uneventful recovery and was discharged on the 4th postoperative day. Intraoperatively she received a single dose of Cefoxitin 1g and Metronidazole 500 mg. Postoperatively, she received the same antibiotic combination for 3 consecutive days. Paracetamol and Tramadol were administered intravenously as analgesics, along with intravenous fluids, for the same number of days. Oral feeding was initiated on the 3rd postoperative day.

Despite the patient and her parents initially denying a history of trichophagia, it was later revealed, upon the first psychiatric consultation, that the patient was exhibiting both trichotillomania and trichophagia. Today, five years after the surgical procedure, she has been attending regular meetings with a psychiatrist.

## 3 DISCUSSION

Bezoars of the GI tract are a rare pathological entity which presents mainly in the stomach. A recent study, extending over a period of more than 20 years, reports a total of 40 cases among 58.000 upper GI endoscopies, representing an incidence of 0.068%.^4^

Interestingly, bezoars can form in any segment of the GI tract.^3^ However, the stomach is the most common organ to be affected.^3^ Bezoars can occasionally occur in individuals with normal GI anatomy and physiology.^1^, ^5^ Nevertheless, patients with altered anatomy or motility of the GI tract are at increased risk of developing them.^1^, ^5^

Several factors can contribute to the formation of bezoars.^3^ Previous gastric surgery appears to be the most common risk factor, as it decreases the size of the stomach, interferes with the passage of gastric contents, and leads to a reduced secretion of gastric acid.^3^ Other predisposing risk factors include diabetes mellitus, autoimmune diseases, peptic ulcer disease, Crohn's disease, carcinoma of the GI tract, hypothyroidism, and excessive fiber intake.^1^ These pathologies can affect the pyloric sphincter's function, normal gastric pH, and gastric motility, leading to delayed stomach emptying.^1^

A trichobezoar is a hair ball trapped in the gastrointestinal tract, affecting mostly females (in approximately 90% of cases) between the ages of 10 and 19 years, but only in half of them, a history of trichophagia is found.^6^, ^7^ The term trichobezoar was first described by Swain in 1854 following postmortem findings, in a patient presenting with acute abdomen.^7^ Human hairs cannot be digested and are retained in the gastric folds, escaping from the peristaltic propulsion^2^, ^7^, ^8^; thus, accumulating in the stomach where they are denatured and oxidized by gastric acid, and combined with food, form an entangled mass.^2^, ^7^, ^8^

The accurate incidence of trichobezoars is unknown. From our review of the literature, we have concluded that this medical condition is not as uncommon as it is referred by several authors. It is difficult to establish how many distinct cases of trichobezoars have been reported, since most of them are published in medical journals which are found in small search platforms. Furthermore, some of these cases are repeated in different publications as case reports, case series, clinical studies, or review articles. The actual number of reported cases must be several hundred.

The size of trichobezoars varies from a small (2 × 2 cm in diameter) to giant size (30 × 15 × 10 cm).^8^, ^9^, ^10^, ^11^ The average size of mostly found trichobezoars is 5.5 cm (mean, 3‐10 cm).^12^, ^13^ The size of trichobezoar in our case was 27 × 10 × 10 cm, which classified it among the giant trichobezoars.

The diagnosis of a trichobezoar involves, firstly, a detailed patient's medical history, focusing primarily on their dietary habits.^2^, ^7^, ^8^ Trichotillomania and trichophagia may be observed in female patients with concurrent psychological or behavioral conditions such as depression and personality disorders.^6^, ^7^ The physical examination, when a trichobezoar is suspected, may reveal severe halitosis and patchy hair loss.^7^ Symptoms may include abdominal pain, a feeling of fullness in the epigastric region, nausea, vomiting, weight loss, dysphagia, or hematemesis; additional symptoms such as hypotension, altered mental state, and shock may develop due to life‐threatening complications such as GI bleeding and obstruction, pancreatitis, necrosis of the visceral wall, and subsequent perforation.^1^, ^2^, ^3^ Blood tests may be indicative of anemia, electrolyte disorders, the presence of trace element deficiencies, hypoproteinemia, and malabsorption of iron and vitamins.^2^, ^7^, ^8^ A well‐defined abdominal mass in the epigastric region is palpable in 85% of patients.^4^ In our patient, the most prominent clinical signs and symptoms were vomiting, abdominal pain, nausea, anemia, and a palpable abdominal mass. The differential diagnosis of a trichobezoar should include other pathological entities such as different types of other bezoars, pancreatic and mesenteric cysts, pancreatic pseudocysts, gastric or pancreatic neoplasms, hydatid disease, volvulus (either cecal or intestinal), and, rarely, ovarian cysts and pregnancy.^14^

An abdominal X‐ray may give rise to clinical suspicion that a trichobezoar is present, while an abdominal U/S depicts the trichobezoar as a non‐vascularized mass of the stomach or other hollow viscus.^1^, ^2^, ^4^, ^7^ In addition, an abdominal CT scan can reveal the presence of a mass and is very helpful in detecting small intestinal trichobezoars.^1^, ^2^, ^4^, ^7^ However, upper gastrointestinal endoscopy remains the diagnostic gold standard and can also be used for treatment in selected cases with small‐sized trichobezoars.^1^, ^2^, ^4^, ^6^, ^7^

Rapunzel syndrome is a rare form of a trichobezoar, with varying definitions. Some authors define the syndrome as a gastric trichobezoar with a tail extending into the jejunum or beyond it, whereas others describe it as a bezoar of any size which may cause intestinal obstruction.^2^, ^8^, ^14^, ^15^ It is a medical condition found mostly in females and from our extensive literature review, only seven cases of males with Rapunzel syndrome have been reported.^16^, ^17^, ^18^, ^19^, ^20^, ^21^ In a systematic review published in January 2020, a total of 110 cases with Rapunzel syndrome were reported by Janssen‐Aguilar et al.^22^

The treatment of a trichobezoar depends on its size, position, and consistency. Endoscopy usually fails to extract the mass of hair, but that depends on its size and density.^1^, ^7^, ^23^ In contrast to phytobezoars, for which Coca‐Cola and other enzymes such as cellulase, pancreatin, papain, and ursodeoxycholic acid have been used successfully for dissolution, trichobezoars are resistant to enzymatic degradation.^24^, ^25^, ^26^

Surgery remains the preferred method of treatment as it offers the possibility of examining the entire gastrointestinal tract and is associated with higher success rates and a low percentage of complications.^9^, ^27^ In small‐ or moderate‐sized trichobezoars, laparoscopy may be used.^28^, ^29^ The laparoscopic removal of giant gastric trichobezoars has been reported only by a few authors.^28^, ^29^ In up to 20% of patients, who have undergone removal of a trichobezoar either surgically or endoscopically, the condition may recur.^30^, ^31^, ^32^ Recurrence may be anticipated when the patient's emotional or psychiatric disorder is not given adequate attention and properly managed through counseling and medication.^30^, ^31^, ^32^

## 4 CONCLUSION

Trichobezoars of the GI tract are a rare medical condition but may pose a potentially serious health risk. It is crucial for physicians to be aware of the risk factors that lead to the development of trichobezoars as well as the subtle clinical findings which may facilitate early clinical investigation and diagnosis. Endoscopy can help reaching the final diagnosis and, in some cases, with the fragmentation and removal of the trichobezoar. Nevertheless, surgery remains the gold standard intervention for the treatment of this medical condition. For patients presenting with trichobezoars, psychiatric surveillance is advised as part of the treatment plan to prevent recurrence by targeting the underlying physical or emotional causes.

## ACKNOWLEDGEMENTS

Not applicable.

## CONFLICT OF INTERESTS

The authors have no potential competing interests or conflicts to report.

## AUTHOR CONTRIBUTIONS

DD, IG, AS, EB, and MG analyzed and interpreted the patient's data. The first draft of the manuscript was written by DD and IG in consultation with KS. All authors commented on previous versions of the manuscript. MF and MC participated in design and coordination. All authors read and approved the final manuscript.

## NAME OF DEPARTMENT AND INSTITUTION WHERE WORK WAS DONE

Department of General Surgery, Venizeleio General Hospital, Crete, Greece.

## ETHICAL APPROVAL

This case report was conducted in accordance with the ethics standards of the institutional and national research committee and with the 1964 Helsinki Declaration and its later amendments or comparable ethics standards. The case report was approved by the Ethics Committee of the Scientific Board of Venizeleio General Hospital of Heraklion (Decision No. 75‐3/Meeting 9/10‐06–2021). Written informed consent was obtained from the patient and her parents. A copy of the written consent is available for review by the Editor‐in‐Chief of the journal.

## CONSENT

Written informed consent was obtained from the patient and her parents for the publication of this case report and any accompanying images. A copy of the written consent is available for review by the Editor‐in‐Chief of the journal.

## Data Availability

The datasets generated and analyzed during the current study are available from the corresponding author on reasonable request.
